# Attention Bias of Avoidant Individuals to Attachment Emotion Pictures

**DOI:** 10.1038/srep41631

**Published:** 2017-01-27

**Authors:** Ying Liu, Yi Ding, Luluzi Lu, Xu Chen

**Affiliations:** 1 Attachment Research Lab, Faculty of Psychology, Southwest University, Chongqing City, 400715, China; 2 Key Laboratory of Cognition and Personality (SWU), Ministry of Education, Chongqing, 400715 China; 3 Huazhong University of Science and Technology, Wuhan, China

## Abstract

How attachment style affects emotion processing is tightly connected with individuals’ attention bias. This experiment explored avoidant individuals’ attentional engagement and attentional disengagement using a cue-target paradigm in fMRI. The experimental group consisted of 17 avoidant participants, while the control group consisted of 16 secure participants; these were identified by the Experiences in Close Relationships inventory and the Relationship Questionnaire. Each reacted to pictures of positive parent-child attachment, negative parent-child attachment, positive romantic attachment, negative romantic attachment, and neutral non-attachment. Behaviorally, avoidant individuals were slower than secure individuals in responding to emotions and their attentional disengagement effect for negative parent-child emotions was stronger than positive ones. fMRI results showed that avoidant compared to secure individuals activated more strongly in the right superior temporal gyrus, middle occipital gyrus, and the left medial frontal gyrus, middle occipital gyrus, supplementary motor area, and cingulate gyrus. They also showed stronger activation in disengaging from positive than negative emotions in the bilateral fusiform and middle occipital gyri. In conclusion, avoidant individuals could detect emotions as effective as secure individuals in attentioal engaging stages. They can disengage from positive emotions with effective cognitive resources and were harder to get rid of negative emotions with insufficient resource.

Attachment style is regarded as a key trait affecting one’s emotional functioning and presentation^1^. Among the three common attachment styles (anxious, secure, and avoidant attachment), anxious individuals show hypervigilance to emotional cues irrespective of emotion valence^2^ and secure individuals exhibit balanced responses to different emotional information^3^,^4^. Avoidant individuals, who are used to inhibiting emotional responses and keeping an emotional distance with others by deactivating emotion processing^5^, however, show higher subjective well-being than anxious individuals^6^ and better cognitive abilities than secure individuals^7^. How can avoidant individuals develop this processing advantage in emotion and cognition? More and more neural evidence suggests that emotion processing of avoidant attachment is closely connected with their attention bias^1^,^8^,^9^.

Attention bias includes two important components of engaging attention with a stimulus and disengaging attention from a stimulus^10^. Various versions of the visual-spatial task have been used to examine them^11^. The classical task involves a cue appearing in one of two locations on a computer screen, with a target appearing either in the cued location or in the uncued location. Reaction time speeds on cued trials reflect attentional engagement with the cue, while slowing on uncued trials reflect costs arising from disengaging attention from the cue. Combined with the fMRI evidence, it has been shown that attentional engagement is an initially automatic process that is connected with the amygdala^12^ and influenced by the superior temporal gyrus^13^. Attentional disengagement is a control process, affected by the anterior cingulate cortex, frontal area, and the dorsal/ventral attention system^12^. However, no direct fMRI evidence has been found to explain attention bias in avoidant attachment.

In ERP studies, one point believed that avoidant individuals’ inhibition of emotional activation was guaranteed by attentional engagement in the early attentional stage. When individuals with three attachment styles watched angry faces, only avoidant individuals had enhanced automatic attentional resources^14^ (the C1 and P1 components^15^) to identify negative emotion^16^. Another experiment also found avoidant individuals had enhanced N170 in response to an emotional face, which indicated more cognitive resources in attentional engegement^17^. However, other ERP studies did not find any special ability of avoidant individuals in emotion processing^18^. When faced with both subliminal and supraliminal presentations, avoidant individuals showed smaller in N100 and N400 than others^19^. N100 is an important indicator of automatic face processing and attention mechanisms; N400 indicates high level semantic processing of the face. The smaller N100 and N400 mean that individuals with avoidant attachment had insufficient attentional resources for automatic emotion perception.

To date, conflicts in ERPs make it necessary to supply new evidence for avoidant individuals’ attention mechanism. Though without direct fMRI studies of avoidantly attached individuals’ attention bias, evidence for automatic processing and control processing connect tightly with the two attentional components, such as those of the primary somatosensory cortex (important automatic neurons), which combine tightly with avoidant individuals’ expression suppression^20^, and the amygdala, which is effective in controlling avoidant individuals’ suppression^21^. Additionally, a much broader network including the thalamus, hippocampus, locus coeruleus, periaqueductal gray, and more were involved in aspects of attention, startle, escape, and avoidance^22^,^23^. With the combination of fMRI evidence in attentional engagement/disengagement and automatic/controlling processing, we can explore how avoidant individuals deactivate emotion information and develop cognitive advantages in the attentional stage.

In addition, the efficacy of emotional cues was an important aspect we must consider. First, attachment-related cues should be chosen rather than non-attachment stimuli, which have been found to be insufficiently effective to distinguish the difference between attachment anxiety and attachment avoidance^24^. Studies have also found that avoidant individuals’ inhibition processing was more effective for attachment-related than non-attachment information^25^. Second, the emotion valence of positive and negative meaning should be chosen as another important aspect in human’s emotional reactions. Attachment relationships of parent-child attachment and romantic attachment were also brought in because of their distinguished social meaning and activating region in the neural study^26^. For example, activity specific to parent-child attachment included regions in the lateral orbitofrontal cortex, the lateral prefrontal cortex, and postero-ventral part of the thalamus^27^, but none of these were activated by romantic love. Therefore, compared with existing studies, it is more suitable and more ecological^28^,^29^ to choose attachment related stimuli of different valences and different attachment themes to investigate emotional functioning in avoidant attachment.

In the present fMRI study, we chose secure individuals as the control group to compare their attentional engagement and attentional disengagement by a cue-target task. We hypothesised firstly that avoidantly attached individuals would show slower emotional reactions behaviorally. Secondly, their deactivating process may be connected with effective activation in automatic brain areas in attentional engagement, such as the superior temporal gyrus and primary somatosensory cortex to negative emotions. Thirdly, they may need more cognitive resources to withdraw from unexpected stimuli in attentional disengagement, with stronger activation in controlling brain areas, such as the orbitofrontal cortex and other frontal areas.

## Method

### Materials

Attachment styles were measured by Bartholomew and Horowitz’s Relationship Questionnaire (RQ), as well as Brennan, Clark, and Shaver’s Experiences in Close Relationships inventory (ECR), whose Chinese versions were both written by Tonggui Li etc^30^. The RQ includes 4 paragraph describing secure attachment (Type A), avoidant attachment (Type B), anxious attachment (Type C), and fearful attachment (Type D). Participants were asked to grade each type on a 7-point scale and choose one most in line with themselves. The ECR has 18 items on attachment anxiety and 18 items on attachment avoidance. Each item describes an experience, which must be judged by the participant for suitability for him- or herself on a 7-point scale ranging from “not at all” to “very much.” ECR is a tool to evaluate individuals’ scores in two dimensions, and is effective in grouping people based on the combination of avoidant and anxious scores. RQ is another self-rating questionnaire with four clear attachment types. Most researches grouped the attachment styles by a single tool. However, ECR was derived from a factor analysis of previously existing measures, such as RQ, AAI(Adult attachment interview) and so on. With the combination of two tools, it can be more effective of selecting the true avoidant group and secure group, which has been used in other attachment research. In Levy’s study of attachment style (2011)^31^, they had integrated the different attachment participants grouping by ECR, RQ and other tools to describe their attachment characteristics. In this study, with the combination of this two tools, it could be more effective and strict to group secure individuals and avoidant individuals.

### Participants

Forty-one undergraduate students took part in the formal fMRI study. All participants were right-handed with normal or corrected-to-normal vision. They had no history of psychiatric illness or neurological problems and none of them had participated previously in a similar study. In this study, one participant was eliminated due to deficiency of behavioral data and seven were eliminated due to head movement. The remaining participants (N_avoidant_ = 17, N_secure_ = 16) were aged 19 to 25 years with a mean age of 22 years. In accordance with the approved guidelines, written informed consent was obtained from the participants prior to conducting pilot or formal experiments. The study was approved by the local Southwest University ethics committee. All methods were carried out in accordance with the approved guidelines.

The selection scores of our sample’s anxiety and avoidance dimensions were compared with the average scores of the whole 300 original questionnaires. For the avoidant group, they should meet type B of the RQ and their avoidant scores should be higher than the average avoidant score of the 300 questionnaires and the anxious score, smaller than that of the 300 questionnaires. For the secure group, they should meet type A of the RQ and their avoidant and anxious scores should be smaller than the average scores of the 300 questionnaires (Table 1). In the secure group, the Pearson’s correlation coefficient of the anxious dimension and avoidant dimension was 0.53 *(P < 0.05). In the avoidant group, the Pearson’s correlation coefficient was 0.52 *(P < 0.05). Before and after the experiment, the State-Trait Anxiety Inventory was used to evaluate individuals’ state anxiety^32^. The pre-test score was 48.74 (SD = 7.74) and the post-test score of was 49.28 (SD = 5.36), which can guarantee that all the participants were in an emotionally stable state.

Image materials consisted of positive romantic attachment, positive parent-child attachment, negative romantic attachment, negative parent-child attachment, and neutral non-attachment pictures. Each kind contains 16 examples to ensure 32 separate trials in the cued condition and uncued condition. Attachment related pictures were selected from the Attachment Affect Picture System (AAPS) by the Attachment Research Lab in Southwest University (China), which had been strictly evaluated along the dimensions of valence, arousal, and attachment from 1 (non-significant) to 9 (very significant); their Cronbach’s alphas all reached 0.90 and split-half reliabilities all reached 0.84 based on 78 raters^33^. This system includes images of negative, positive, and neutral scenes of family leisure, outdoor recreation, sports, daily life, and work life. All images were unified into 96 × 96 dpi resolution, 24-bit depth, and size of 433 × 433 mm^2^.

### Procedure

**Figure 1:**
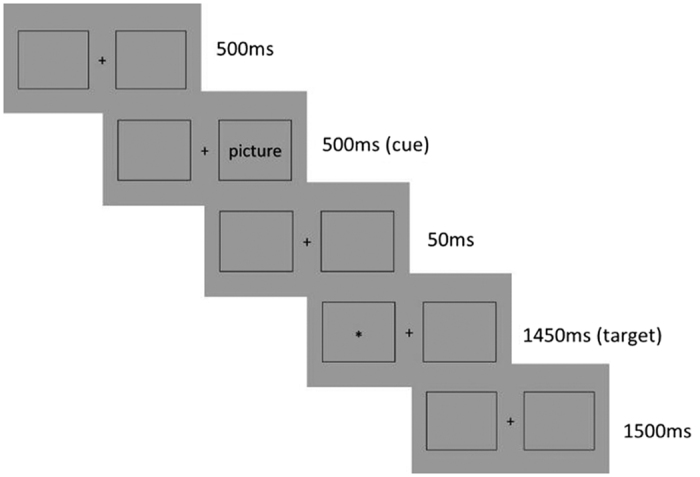
The flow diagram of the experiment.

The cue-target paradigm was used in this study. All compound stimuli were programmed and presented using E-Prime 2.0 on a Dell 19-inch monitor. Participants were lying in a Siemens 3 T scanner with dim light 80 cm from the screen. The viewing angle of the framed body-word compound stimuli on the screen extended 9.87° vertically and 6.58° horizontally. Each trial began with a 500 ms blank screen followed with the picture cue in the left or right on the screen randomly for 500 ms. Then the same blank screen appeared for 50 ms with the target “_*_” followed either in the cued location or in the alternative location randomly for 1450 ms. After that, participants needed to judge the location of the target in 1500 ms (Fig. 1). Every trial lasted 4000 ms as presented above and all participants finished 320 trials. The whole experiment was separated into two eleven-minute runs with one-minute rest in-between. Location difference was balanced in the left and right field of vision.

### Behavioral data analysis

Behavioral statistical analyses were performed using SPSS 16.0 (Statistical Packages for the Social Sciences, Version 16.0, SPSS Inc., USA) with a level of significance of p < 0.05. We deleted data for wrong reactions and reaction times less than 100 ms or longer than 1000 ms. We performed repeated ANOVAs of 2 (attachment group) × 2 (cue validity) × 3 (emotion valence) and 2 (attachment group) × 3 (valence) × 2 (attachment theme). The operational definitions of attentional components were defined to produce in each case a positive score^13^:


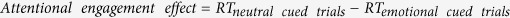



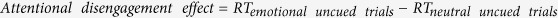


### fMRI data acquisition and analysis

Images were acquired with a Siemens 3 T scanner (Siemens Magnetom Trio TIM, Erlangen, Germany). An echo-planar imaging (EPI) sequence was used for data collection, and T2-weighted images were recorded per run (TR = 2000 ms; TE = 30 ms; flip angle = 90°; field of view (FOV) = 220 × 220 mm^2^; matrix size = 64 × 64; 32 interleaved 3-mm thick slices; in-plane resolution = 3.4 × 3.4 mm^2^; interslice skip = 0.99 mm). T1-weighted images were recorded with a total of 176 slices at a thickness of 1 mm and in-plane resolution of 0.98 × 0.98 mm^2^ (TR = 1900 ms; TE = 2.52 ms; flip angle = 90°; FOV = 250 × 250 mm^2^). We used SPM8 (Welcome Department of Cognitive Neurology, London, UK, http://www.fil.ion.ucl.ac.uk/spm/software/spm8/) to preprocess the functional images^34^. Slice timing correction was used to correct slice order, the data were realigned to estimate and modify the six parameters of head movement, and the first six images were discarded to achieve magnet-steady images. These images were then normalized to Montreal Neurological Institute (MNI) space in 3 × 3 × 3 mm^3^ voxel sizes. The normalized data were spatially smoothed with a Gaussian kernel; the full width at half maximum (FWHM) was specified as 4 × 4 × 4 mm^3^.

Then we obtained six direction parameters of the head moving; we deleted the data of participants whose heading moving parameters were over 2.5 mm. After the preprocessing, data on 7 participants were deleted and on 33 participants were retained: that is, the final sample consisted of 17 avoidant individuals and 16 secure individuals of which 14 are females and 19 are males.

Two levels of ANOVA procedures were used to deal with fMRI data. At the first (subject) level, six event types were defined. These consisted of positive cued trials, negative cued trials, neutral non-attachment cued trials, positive uncued trials, negative uncued trials, and neutral non-attachment uncued trials. The onset time was chosen when the target pictures were presented. At the second (group) level, T-tests were chosen for comparison. The contrast images (neutral cued trials-emotion cued trials for attentional engagement, emotion uncued trials-neutral uncued trials for attentional disengagement) from two groups were the input data. To determine whether there was significant activation corresponding to each contrast, a corrected *p* = 0.05 and extent threshold of *cluster size* = 20 voxels for the height (intensity) were used as the threshold.

## Results

### Behavioral data

**Figure 2:**
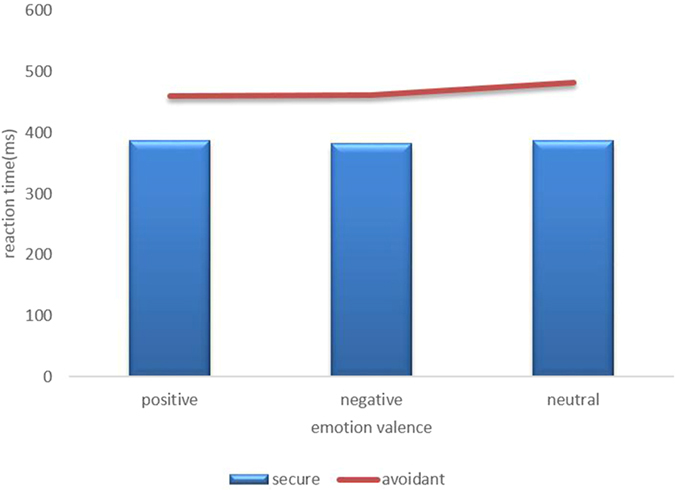
RTs(ms) of two groups to positive, negative and neutral pictures (*P *< 0.05).

In repeated measures ANOVA of 2 (group) × 2 (cue validity) × 3 (emotion valence), a significant main effect of cue validity was observed (F_1,31_ = 38.29; p < 0.001); a significant main effect of emotion valence was observed (F_2,62_ = 10.49; P < 0.01); the interaction of cue validity and attachment style reached significance (F_2,62_ = 4.25; p < 0.05) (Table 2 and Fig. 2).

Attentional engagement and disengagement were analyzed by repeated ANOVA of 2 (attachment style) × 3 (valence). Testing attentional engagement in the cued situation, the main effect of valence reached significance (F_2,62_ = 8.20; p < 0.01), the attentional engagement effect of positive emotion was 10.73 ms (p < 0.01) and the attentional engagement effect of negative emotion was 11.57 ms (p < 0.01). The difference between the two groups did not reach significance. Testing attentional disengagement in the uncued situation, the main effect of valence reached significance (F_1,31_ = 5.24, p < 0.05). Further data showed the RT of neutral (448.98 ms) was slower than positive emotion (439.08 ms) and negative emotion (435.17 ms), which means they did not show attention disengagement to attachment emotion.

When considering different emotion themes of parent-child and romantic images in the cued situation, no attentional engagement effect was found. In the uncued situation, the repeated ANOVA of 2 (group) × 3 (valence) × 2 (attachment theme) showed that the main effect of valence reached significance (F_2,62_ = 4.23; p < 0.05); the main effect of theme also reached significance (F_2,62_ = 6.85; p < 0.05); the interaction of attachment styles × valence × themes reached significance, F_2,62_ = 3.56, p < 0.05. Testing the simple effect of emotion valence, the attentional disengagement effect of avoidant individuals for negative parent-child images was 7.08 ms (p < 0.05) and the attentional disengagement effect of secure individuals for positive parent-child images was 10.25 ms (p < 0.05). Testing the simple effect of attachment themes, attentional disengagement of secure individuals for positive parent-child images was 17.07 ms (p < 0.05). Attentional disengagement of avoidant individuals for negative parent-child images was 15.24 ms (p < 0.05).

As shown in Table 3 below, significant correlations of the two attentional components in different emotions existed in both the secure and avoidant groups.

## Imaging data

### Group analysis

**Figure 3:**
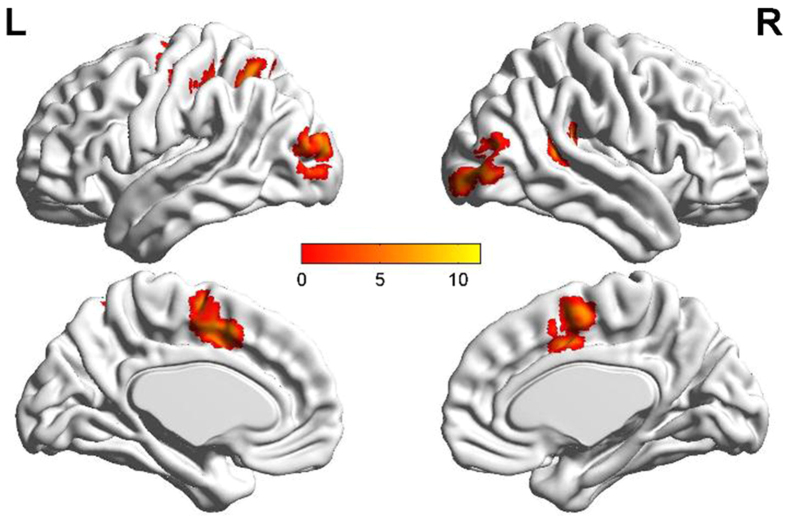
Group analysis of aovidant group > secure group (FWE = 0.05, cluster voxel = 20).

When we compared the group effects, the contrast of avoidant group to secure group revealed significant activation in the whole-brain analysis. Avoidant individuals showed stronger activation in the right superior temporal gyrus, middle occipital gyrus, and the left medial frontal gyrus, middle occipital gyrus, supplementary motor area, and cingulate gyrus than secure individuals (FWE = 0.05, cluster size = 20) (Fig. 3 and Table 4).

**Figure 4:**
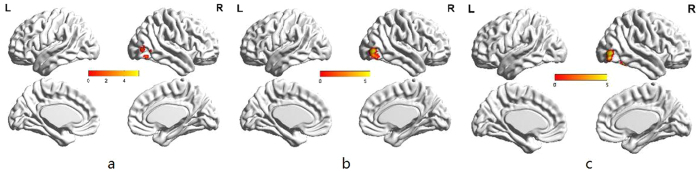
Separate presentation of secure individuals’ attentional engagement to negative emotion . (**a**), attentional disengagement to negative emotion (**b**) and attentional disengagement to positive emotion (**c**) (FWE = 0.05, cluster voxel = 20).

When analyzing the attentional engagement of secure individuals, significant activation in the right fusiform gyrus (x = 45–66, y = −54–66, z = 3–15) and the middle occipital gyrus to negative emotion (peak voxel coordinate, x = 48–54, y = −75, z = −3–0,) were found, but not to positive emotion (FWE = 0.05, cluster size = 20). Their activation of disengagement was close to the response of engagement to negative emotion (Fig. 4).

**Figure 5:**
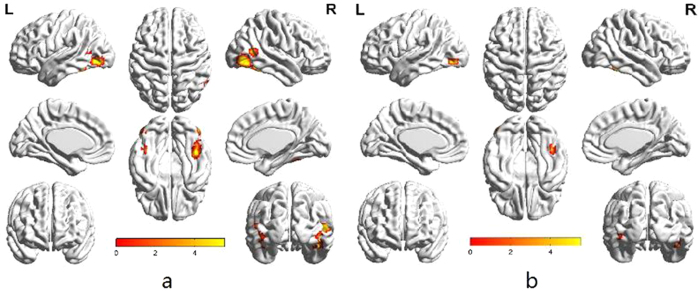
Separate presentation of avoidant individuals’ attentional engagement to negative emotion (**a**) and attentional engagement to positive emotion (**b**) (FWE = 0.05, cluster size = 20).

When analyzing the attentional engagement of avoidant individuals, bilateral activation in the fusiform gyrus and right activation in the middle occipital gyrus were found both to positive and negative emotion (FWE = 0.05, cluster size = 20) (Fig. 5 and Table 5).

**Figure 6:**
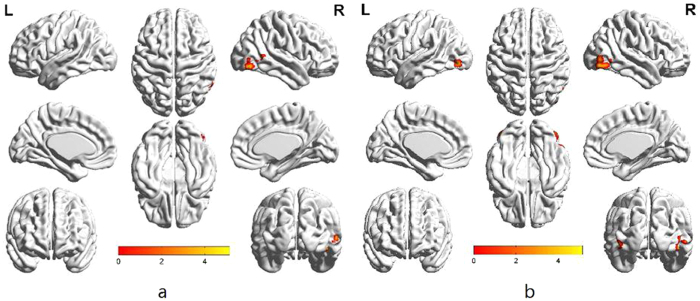
Separate presentation of avoidant individuals’ attentional disengagement to negative emotion (**a**) and attentional disengagement to positive emotion (**b**) (FWE = 0.05, cluster size = 20).

When comparing the attentional disengagement of avoidant individuals, we found bilateral activation in the fusiform gyrus and the middle occipital gyrus to positive emotion but not to negative emotion (FWE = 0.05, cluster size = 20) (Fig. 6).

## Discussion

This is the first study to investigate attentional components of avoidant individuals’ emotion bias by fMRI. Behaviorally, avoidant individuals were slower than secure individuals in responding to emotion, which confirmed our first hypothesis. fMRI results showed that avoidant compared to secure individuals had stronger activation in the right superior temporal gyrus, middle occipital gyrus, and left medial frontal gyrus, middle occipital gyrus, supplementary motor area, and cingulate gyrus. These areas connected with their early attentional engagement and confirmed our second hypothesis that early automatic processing may guarantee avoidant individuals’ deactivation. Avoidant individuals’ stronger activation in disengaging from positive than negative emotion in the bilateral fusiform and middle occipital gyri, also supplemented our third hypothesis of their special attention bias. Based on these findings, we concluded that avoidant individuals’ attentional engagement was tightly connected with their automatic processing. Their disengagement effect was stronger in processing negative than positive stimuli and their success in disengaging from positive emotion was based on their efficient emotional activation. These findings provide a new view of avoidant individuals’ attention bias and inspire us with a new viewpoint to research adult attachment.

### Group differences in attention bias

Avoidant individuals were significant slower than secure individuals, which was associated with their deactivating process and unsocial interpersonal relationship^35^ revealed in previous studies^15^,^17^,^25^. However, deactivation does not mean weak neural processing. Avoidant individuals even had stronger activation in the superior temporal gyrus, cingulate gyrus, supplementary motor area, and middle frontal gyrus than secure individuals. The superior temporal gyrus relates to automatic alerting reactions and the middle frontal gyrus relates to executive control reactions^13^. Their enhanced activation in the avoidant group means more attentional resources were being used. The cingulate gyrus is tightly connected with attachment emotion, especially for negative emotions^36^. Though the cingulate gyrus of avoidant individuals was less activated than for anxious individuals^37^, the stronger activation than secure individuals here could also confirm that avoidant individuals may be more sensitive in processing attachment emotion stimuli, which is also consistent with the stronger C1 and P1 in Dan’s study^15^.

As an important area associated with automatic actions, stronger activation of the supplementary motor area may be explained as more effective attentional engagement to attachment emotion^38^. However, the finding of the supplementary motor area (BA3/4) was inconsistent with the activation of the somatosensory cortex (BA3) in Suslow’s study^20^. They found that attachment avoidance was inversely related to responses of the primary somatosensory cortex (BA3) to masked sad faces and explained it could be their habitual unwillingness to deal with needs for proximity, especially for highly avoidant individuals. However, combined with more executive control resource of the middle frontal gyrus in our finding, this may be explained in that attentional engagement needs automatic brain responses to achieve emotional recognition to guarantee avoidant individuals’ deactivation, such as shifting their attention away from attachment-related stimuli (e.g., pictures of one’s mother, pictures of people separating^39^) to promote the completion of deactivation. It may reflect avoidant individuals’ insufficient in balancing automatic process and control process, as what we found in the correlations of attentional engagement and disengagement.

In both groups, their attentional engagement and disengagement separately showed significant positive correlations between negative and positive emotion, which means individuals’ attentional components remain stable in different emotions. However, significant negative correlations were found in the avoidant group. Their negative correlations mean that, if avoidant individuals have enhanced attentional alerting to positive emotion, they may have weak attentional withdrawal from negative emotions. If they have enhanced attentional alerting to negative emotion, they may also have weak attentional withdrawal from negative emotions. As Edelstein^25^ found that avoidant individuals can inhibit attention to potentially threatening information, but this ability requires cognitive effort^25^. Although avoidant individuals have advantages in dealing with unemotional activities, their insecure attachment may weaken the cognitive ability in balancing automatic emotional process and control emotional process than secure individuals, especially when facing with emotion of parent-child relationship. Stronger neural activation of avoidant individuals and slower behavioral responses seem contradictory, but this may represent that effective automatic neural activities guarantee avoidant individuals’ attentional engagement and ineffective coordination of controlling process affect their shifting of attention with a longer reaction time.

### Valence difference of avoidant individuals

The fusiform gyrus was tightly connected with attentional bias and detection of emotional information^40^. The higher one’s detection level reaches, the stronger activation of the fusiform gyrus becomes. As an important area of face recognition^41^, it was also a special area for attachment emotion, which is tightly connected with attachment information (familiar faces)^42^. In the present study, both groups showed stronger attentional engagement by stronger activation of the right fusiform and middle occipital gyrus to negative than positive emotion, which was consistent with the idea that negative attachment emotion can engage more attention resources without respect to attachment styles^43^.

However, when comparing the attention disengagement of avoidant individuals, bilateral activation occurred in the fusiform and middle occipital gyrus to positive but not to negative emotion, which implies that avoidant individuals need more resources to identify emotion characteristics and complete the separation them from positive emotion. Positive emotions were more unexpected for avoidant individuals, and once other information was engaged in consciousness, this unfamiliarity needed more activation to complete their shifting of attention. Interestingly, though no behavioral differences were found in two groups’ attention bias, when considering attachment relationships (parent-child), avoidant individuals showed difficulty disengaging from negative parent-child emotion and secure individuals showed difficulty disengaging from positive parent-child emotion. Avoidant attachment is developed from distant or slighted interaction with their care-takers. This finding can provide effective evidence that negative situation, as a reminder of dangerous, can prompt avoidant individuals to distinguish parent-child attachment and romantic attachment, which also confirms that avoidant individuals show attention bias to negative emotions, especially for those rooting in their childhood.

There remain unresolved issues in the current study. In group level, although comparation of avoidant and secure attachment were discussed, characteristics of different avoidant subtypes, such high avoidant attachment and low avoidant attachment, need to be studied in later experiments. In neural level, are identical brain structures responsible for attention and emotion conflict with variation in different styles, or are both common and dissociable brain areas involved in dealing with different types of emotion based attentional engagement and disengagement ? Does a mixed effect of valence, theme and cue validity existing in individuals’ attentional response? Further studies are needed to resolve these issues. In summary, the present study provides evidence that attention bias is relevant in discussing avoidant individuals’ emotional functioning and addresses the importance of considering attentional components and attachment-related stimuli when exploring attachment style’s influence in emotional processing.

## Additional Information

**How to cite this article**: Liu, Y. *et al*. Attention Bias of Avoidant Individuals to Attachment Emotion Pictures. *Sci. Rep.* **7**, 41631; doi: 10.1038/srep41631 (2017).

**Publisher's note:** Springer Nature remains neutral with regard to jurisdictional claims in published maps and institutional affiliations.

## Figures and Tables

**Table 1:** ECR scores, RQ types, and others descriptive statistics of the two groups.

	Total	Avoidant group	Secure group	t	p
N	33	17 (female = 6)	16 (female = 8)		
Age	21.69 ± 2.05	21.35 ± 0.56	21.87 ± 0.45	0.48	0.64
Anxious score	2.94 ± 0.64	3.07 ± 0.18	2.81 ± 0.12	−1.22	0.23
Avoidant score	3.77 ± 1.20	4.79 ± 0.12	2.68 ± 0.14	−11.39	0.00**
RQ		B	A		

**p < 0.001.

**Table 2:** RTs of two group to positive, negative, and neutral pictures (ms).

	Positive	Negative	Neutral
Secure	387.53 ± 38.26	382.02 ± 38.43	386.80 ± 29.96
Avoidant	460.65 ± 40.36	461.59 ± 43.03	482.70 ± 42.53

**Table 3:** Correlation of two attentional components in different emotions of the secure and avoidant groups.

		Engagement of P	Engagement of N	Disengagement of P
Secure	Engagement of P	—	0.76**	—
	Disengagement of N	—	—	0.94**
Avoidant	Engagement of P	—	0.70**	—
	Disengagement of N	−0.55*	−0.53*	0.89**

*p < *0*.05; **P < 0.01. P means positive emotion; N means negative emotion.

**Table 4:** Areas of significant activity for the contrast between avoidant individuals compared to secure individuals.

Region	BA	X	Y	Z	Z-score	Voxel
**R Superior Temporal Gyrus**	22	42	−39	12	4.52	76
**L Medial Frontal Gyrus**	6	−12	−3	60	4.33	155
**L Middle Occipital Gyrus**	19	−30	−96	12	3.59	52
**R Middle Occipital Gyrus**	19	45	−81	0	6.47	91
**L Supp. motor Area**	3/4	−38	−21	45	4.46	77
**L Cingulate Gyrus**	32	3	4	43	4.89	97

**Table 5:** Avoidant individuals’ attentional engagement to negative emotion.

Region	BA	X	Y	Z	Z-score	Voxel
R Fusiform	37	42	−42	−21	3.88	65
R Middle Occipital Gyrus	19	45	−75	−3	4.21	50
R Angular gyrus	39	48	−57	9	5.64	40
L Fusiform Gyrus	37	−42	−48	−21	5.07	25
L Superior Temporal Gyrus	22	−51	−60	12	6.49	35
